# Ultraprocessed Food is Not a Replacement for Whole Food

**DOI:** 10.1016/j.advnut.2023.05.016

**Published:** 2023-06-02

**Authors:** Owen J. Kelly

**Affiliations:** Sam Houston State University College of Osteopathic Medicine, Department of Molecular and Cellular Biology, Conroe, TX, United States

Dear Editor

Regarding the recently published article by Valicente et al., [1] entitled “Ultraprocessed foods and obesity risk: a critical review of reported mechanisms,” the NOVA classification system is not perfect; however, its foundation in nutritional science and its good intentions for the wellbeing of the citizens of the world is not in question. The guiding principle of NOVA—to consume more whole foods—is fundamental in other dietary guidelines [2]. The goal must be to improve NOVA and integrate it into broader dietary guidelines.

The main focus is related to your suggestion that ultraprocessed foods are not lacking in nutrients and can contribute to achieving micronutrient targets. This is true in principle; however, adding micronutrients, or some other nutrient, such as fiber, to ultraprocessed foods should not automatically make the food ‘healthy’; of course, it depends on one’s definition of ‘healthy,’ which is within itself an issue. Nevertheless, the foods recommended by dietary guidelines are whole foods, and many do not even need food labels. Therefore, healthy eating patterns, good dietary quality, and whole foods are the cornerstone for preventing and treating disease. However, robust randomized clinical trial data are lacking to absolutely support any recommendation, including Dietary Guidelines for Americans (DGA), other guidelines [2], or NOVA. Over all the decades of DGA and dietary reference intakes (DRI), there has never been a randomized, controlled, clinical trial to investigate outcomes from a diet that contains all the nutrients at their DRI level, as compared with one with lower (or higher) levels.

The outdated view of nutrition and public health is to consume enough of each nutrient to prevent deficiency; public health policy and food manufacturers have followed this for decades. Public health policy is changing (see current DGA); however, food manufacturers seem slower to react to the need to include more whole foods in the diet [3]. Nutritional science, over the last few decades, has been entrenched in this reductionist approach; however, it is slowly changing [4].

Of course, fortified or enriched ultraprocessed foods will help achieve the current, relatively soft, nutritional targets (eg, daily values). In this case, then, what you may be describing in your paper [1] is that *fortified ultraprocessed foods may help to prevent deficiencies of essential nutrients in a diet low in whole foods*. Fortification programs have had public health successes, especially the addition of folic acid to wheat flour. Why fortify with folic acid—because the diet was low in that nutrient, due to lower whole food intakes; the best sources of folate are fruits, leafy green vegetables, and legumes.

The fundamental question is then, what makes whole foods different from ultraprocessed foods? Valicente et al. [1] may lead one to believe that there is no difference—ultraprocessed foods do have essential nutrients. However, the essential nutrients are the tip of the iceberg as it relates to the number and variety of compounds in whole foods. For example, garlic, historically a medicinal plant, has ∼2306 known compounds, yet 37 compounds are focused on for health messages [5]; there is no focus on the macronutrient or micronutrient composition. However, interactions with some, or all, of the other 2269 compounds may be as important for achieving the beneficial outcomes from whole garlic. The potential for these other compounds as it relates to health benefits has been referred to as the “dark matter of nutrition” [6]. FooDB (www.foodb.ca) is working to identify and track these compounds. Although many of the compounds are not currently associated with deficiency disease or with health benefits, it cannot be denied that the search for functional foods and nutraceuticals is ultimately focused on the *nutritional dark matter* of whole foods [7,8], which may or may not be present in ultraprocessed foods. It is important to note, the other layers of complexity are related to how processing, cooking, metabolism, or gut bacteria change the properties of these *nutritional dark matter* compounds.

Humans evolved within an environment that includes whole foods, including the *nutritional dark matter*. It is safe to hypothesize that with whole foods, the metabolome is much more complex and possibly beneficial. One could speculate that what is missing from the modern diet, in relation to our evolutionary food environment, is the *nutritional dark matter*. This reveals a very large knowledge gap in human nutrition. Although both sides of the NOVA debate can use the phrase “absence of evidence is not evidence of absence” to demonstrate their point, it seems the larger burden of proof relates to how the human metabolome has changed as it transitioned from whole foods to ultraprocessed foods and the health consequences of it. Ideally the burden of proof should be on the manufacturers of ultraprocessed foods. Regardless, research into the effects of whole foods on the metabolome and health certainly deserves attention.

One point about cost, the cost of whole foods is absolutely an issue. In a large US retailer, a 48-oz jar of apple sauce was $2.96, whereas the same weight of apples cost $4.84 (on sale). Whole apples, which require little processing, should cost less than apple sauce—there is something amiss.

Ultraprocessed foods are not “bad” per se; no single food item is bad for you. However, applying the toxicology principle, attributed to Paracelsus, “solely the dose determines that a thing is not a poison,” may explain why ultraprocessed foods are negatively associated with health. This also agrees with the idea to consume all foods in moderation; however, ultraprocessed foods should probably not be substitutes for whole foods. Whole foods shold also be consumed in moderation, e.g., pears contain small amounts of formaldehyde. Based on the missing *nutritional dark matter*, the premise of limiting ultraprocessed foods should not be in question. Nutritional scientists, and others, must be allowed to create food policy and guidelines with the sole purpose of having a healthier population. Ideally, this should be without any external political or commercial interests. However, nutritional science has much to learn and must evolve, but first it must leave behind the shackles of the past.

## Funding

The author reported no funding received for this study.

## Author disclosure

The author reports no conflicts of interest.
